# In Situ Hybridization of PRRSV-1 Combined with Digital Image Analysis in Lung Tissues of Pigs Challenged with PRRSV-1

**DOI:** 10.3390/vetsci8100235

**Published:** 2021-10-15

**Authors:** Lilla Dénes, Dávid G. Horváth, Oliver Duran, Poul H. Ratkhjen, Christian Kraft, Balazs Acs, Attila M. Szász, Till Rümenapf, Marton Papp, Andrea Ladinig, Gyula Balka

**Affiliations:** 1 Department of Pathology, University of Veterinary Medicine, István u. 2, 1078 Budapest, Hungary; denes.lilla@univet.hu (L.D.); horvath.david.geza@univet.hu (D.G.H.); 2 Boehringer Ingelheim Vetmedica GmbH, 55218 Ingelheim am Rhein, Germany; oliver.duran@boehringer-ingelheim.com (O.D.); poul.rathkjen@boehringer-ingelheim.com (P.H.R.); christian.kraft@boehringer-ingelheim.com (C.K.); 3 Department of Oncology and Pathology, Karolinska Institutet, CCK R8:04, 17176 Stockholm, Sweden; balazs.acs@ki.se; 4 Clinical Pathology and Cancer Diagnostics, Karolinska University Hospital, Building 70, Level-2, 11883 Stockholm, Sweden; 5 Department of Internal Medicine and Oncology, Semmelweis University, Korányi Sándor u. 2/a, 1083 Budapest, Hungary; szasz.attila_marcell@med.semmelweis-univ.hu; 6 Institute of Virology, Department of Pathobiology, University of Veterinary Medicine Vienna, Veterinaerplatz 1, 1210 Vienna, Austria; till.ruemenapf@vetmeduni.ac.at; 7 Centre for Bioinformatics, University of Veterinary Medicine, István u. 2, 1078 Budapest, Hungary; papp.marton@univet.hu; 8 University Clinic for Swine, University of Veterinary Medicine Vienna, Veterinaerplatz 1, 1210 Vienna, Austria; andrea.ladinig@vetmeduni.ac.at

**Keywords:** porcine reproductive and respiratory syndrome virus, RNAscope, in situ hybridization, qRT-PCR, QuPath

## Abstract

*Betaarterivirus suid 1* and *2* are the causative agents of porcine reproductive and respiratory syndrome (PRRS), which is one of the most significant diseases of the swine industry, causing significant economic losses in the main pig producing countries. Here, we report the development of a novel, RNA-based in situ hybridization technique (RNAscope) to detect PRRS virus (PRRSV) RNA in lung tissues of experimentally infected animals. The technique was applied to lung tissues of 20 piglets, which had been inoculated with a wild-type, highly pathogenic PRRSV-1 strain. To determine the RNAscope’s applicability as a semi-quantitative method, we analysed the association between the proportion of the virus-infected cells measured with an image analysis software (QuPath) and the outcome of the real-time quantitative reverse transcription polymerase chain reaction (qRT-PCR) tests performed in parallel. The results of the quantitative approach of these two molecular biological methods show significant association (pseudo R^2^ = 0.3894, *p* = 0.004). This is the first time RNAscope assay has been implemented for the detection of PRRSV-1 in experimental animals.

## 1. Introduction

Porcine reproductive and respiratory syndrome is one of the most widespread and economically devastating diseases in the global swine industry [1,2]. PRRS virus (PRRSV) comprises two species *Betaarterivirus suid 1* and *2* belonging to the *Betaarterivirus* genus within the *Arteriviridae* family of the *Nidovirales* order [3]. PRRSV is characterized by a high degree of genetic diversity and variability between and within the two species [4,5]. The virus’ primary target organ is the lung, where it replicates predominantly in alveolar macrophages, but has been identified in intravascular and interstitial macrophages as well [6,7,8,9]. The hemoglobin/haptoglobin scavenger receptor CD163 has been identified as the primary receptor for virus entry into the target cells, as genome-edited animals with a knock-out of either the entire CD163 or just the virus interaction site were resistant to infection [10,11]. Clinical symptoms of PRRS can be very diverse, ranging from asymptomatic infections to outbreaks of high fever and hemorrhagic disease with high morbidity and mortality. Reproductive disorders, abortions, preterm farrowings, and the delivery of stillborns are typical in sows and gilts, and the infected piglets are born with decreased weight and vitality. Affected piglets and fatteners show respiratory symptoms and, in boars, the semen quality can deteriorate [12,13].

Polymerase chain reaction after reverse transcription (RT-PCR) is the most common method for the direct detection of PRRSV. Viral quantity can be examined with the widely used real-time quantitative PCR, however, the exceptional genetic diversity of the strains might affect the sensitivity of the different methods [14,15]. Identification and visualization of a virus in tissues can be performed by detecting its antigens with immunohistochemistry (IHC) or fluorescent antibody (FA) staining or its nucleic acids by in situ hybridization (ISH). The common advantage of these methods over PCR is that they can identify the pathogen within the lesions. These methods, however, are usually less sensitive than PCR owing to genetic and antigenic variability of the target organism and autolysis or improper fixation, and tissue processing can also significantly decrease the detection sensitivity of these assays. In the case of PRRSV, antigen detection with antibodies raised against the nucleocapsid protein (N) or the glycoprotein 5 (GP5) can be performed by chromogenic IHC on formalin fixed paraffin embedded (FFPE) tissues, or by FA staining on frozen slides. However, these techniques are considered less sensitive than viral nucleic acid detecting methods [16], most likely because of the high degree of antigenic variability among the recent strains and the few commercially available antibodies. In situ hybridization assays target specific nucleic acid sequences using a probe, complementary to the genome of the pathogen. Typically, digoxigenin, biotin, or dinitrophenol (DNP) is used to label the probe. As a general disadvantage, owing to lower labeling efficiency and decreased target size, oligonucleotides used in classical in situ hybridization assays are less sensitive than RNA probes (riboprobes) [17]. Larochelle et al. developed an in situ hybridization assay based on the digoxigenin labeled cDNA probe for the detection of PRRSV in cell cultures and FFPE tissues. The authors considered ISH to be a sensitive and specific method for the diagnostics of PRRS as well as a useful tool in retrospective and pathogenesis studies [18].

Recently, a novel, RNA-based ISH technology (RNAscope^®^, Advanced Cell Diagnostics Inc., Biotechne, Abingdon, UK) has been developed and introduced. The method uses Z shaped, 18–25 base long pairs of RNA probes that are designed to bind next to each other on the template RNA. A positive fluorescent/chromogenic signal is generated only if the two probes hybridize side by side and are thus able to bind the L-shaped, labeled amplifier probe on their opposite side. This structure ensures the high specificity and sensitivity of the procedure, as the bond of three probe pairs only is sufficient to result in a visible positive signal. As these hybridization oligonucleotides bind to only 18–25 base long segments of the RNA, partially damaged RNA can also be detected by the RNAscope method [19,20,21]. Owing to the abovementioned properties of the assay, it can be used in relatively decomposed samples, and the genetic variability of the targeted gene will not reduce its sensitivity as much as in the case of conventional ISH techniques deploying single, linear oligonucleotide probes. The technique has already been used to detect various porcine viruses including Seneca valley virus [22], a neuroinvasive astrovirus [23], and PCV3 [24], and our research group has recently used it to detect the atypical porcine pestivirus [25].

Digital slides and software-based image analysis are widely used techniques in experimental and diagnostic human pathology. Image analysis software is capable of objective digital cell countings and staining intensity measurements, which can replace the difficult, fastidious, and time-consuming manual cell counting, which is often hard to reproduce and shows significant inter-observer variability [26].

The first objective of this study was to develop an ISH technique to detect PRRSV in tissue samples from a highly virulent PRRSV-1 strain respiratory challenge model in young pigs. Second, we aimed to perform digital image analysis and software based ISH positive cell counting and to investigate the association between these results and the routine histological pneumonia severity scores as well as the qRT-PCR data obtained from samples of the same lung lobes.

## 2. Materials and Methods

### 2.1. Animal Ethics Approval

The entire experiment was carried out according to the current Hungarian animal welfare regulations, under the ethical permission number: BA02/2000-43/2017.

### 2.2. Sample Collection

Altogether, 25 seven-weeks-old pigs were included in the study, 20 of which were inoculated intranasally with 2.5 mL of cell culture supernatant containing 10^6^ TCID_50_ of the highly virulent Austrian PRRSV-1 isolate AUT15-33, also known as “ACRO” [27]. Five pigs were sham inoculated, serving as negative controls. All pigs used in the experiment were humanely euthanized on the 14th DPI at 9 weeks of age. During necropsy, selected organs, including every lung lobe from both lungs, were sampled and immersed in 10% neutral buffered formaldehyde (NBF) solution for histopathological analysis and in situ hybridization. Match lung lobe samples were sent in parallel to the University of Veterinary Medicine in Vienna for PRRSV viral RNA quantification.

### 2.3. Tissue Processing and Routine Histology

After 24 h of fixation at room temperature in the formaldehyde solution, samples were trimmed and dehydrated with series of ethanol and xylene in an automatic tissue processor. The dehydrated tissue samples were embedded in paraffin blocks and 4 µm thin sections were cut manually and mounted onto Superfrost+ adhesion slides (Thermo Fisher Scientific, Waltham, MA, USA).

The unstained sections were deparaffinized and rehydrated in xylene and alcohol, respectively. Routine hematoxylin and eosin staining was performed in an automatic stainer instrument.

Lesion severity and distribution were scored according to Balka et al. [28]. Briefly, the changes evaluated included (1) pneumocyte hypertrophy and hyperplasia, (2) septal mononuclear infiltration, (3) intra-alveolar necrotic debris, (4) intra-alveolar inflammatory cell accumulation, and (5) perivascular inflammatory cell accumulation. The lesions were scored for severity (0–3) and distribution (0–3) in all seven lung lobes and they were added up to obtain a score for the separate lobes and for the overall lungs (the maximum score was 30 for a lobe and 210 for the entire lungs).

### 2.4. RNA-Based In Situ Hybridization—RNAscope

The viral specific RNAscope probe (Cat.No. 519,571) was designed based on the sequence of AUT15-33 (Acc. no.: MT000052.1), targeting the ORF7 region of the viral genome. Probes targeting the mRNA of the ubiquitous, widely expressed housekeeping gene peptidyl-prolyl-isomerase-B (*Sus scrofa*-PPIB, Cat.No. 428591)) were used as positive control, while probes targeting bacterial dihydropicolinate reductase (DapB, Cat.No. 310043)) were used as a negative control.

The ISH process was performed on the left medial lobe of the animals’ lungs. This lobe was selected to overcome the possible effect of different lesion distribution (the cranial and middle lobes invariably displayed more severe lesions than the caudal lobes). The assay was performed according to the manufacturer’s protocol. After blocking the endogenous peroxidase activity, the slides were boiled in the previously prepared 1X Target Retrieval solution for 15 min and washed twice with distilled water, followed by washing in 96% ethanol. After removal from ethanol, the samples were air-dried at room temperature. RNAscope^®^ Protease Plus solution was added and the slides were incubated in a hybridization chamber (HybEZ™ Oven, Advanced Cell Diagnostics, Newark, CA, USA) for 30 min at 40 °C.

During the next step, 200 µL of the respective hybridization probes was added to each sample. The samples were incubated at 40 °C for 2 h in a hybridization thermostat (HybEZ™ Oven). After the incubation time, the sections were washed twice in the previously prepared 1X wash buffer solution for 2 min at room temperature. After draining the excess liquid, amplification reagents (Amp-1–6) were sequentially added to the samples.

Thereafter, sections were treated with approximately 120 µL of a 1:60 mixture of Fast RED-B and Fast RED-A solutions and incubated for 10 min in the hybridization chamber at room temperature. After washing with distilled water, sections were placed in Hematoxylin Solution, Gill No. 2 (Merck, Darmstadt, Germany) solution for 2 min. This was followed by washing twice with distilled water, placing samples in 0.02% ammonia solution for 10 s, then washing again with distilled water, and finally drying at 60 °C for 15–25 min. After drying, the slides were immersed in xylene, EcoMount medium was spotted onto them, and they were covered with cover slips.

### 2.5. Section Scanning and Software Analysis

One section per piglet (left medial lobe) treated with the RNAscope method was subsequently scanned and digitalized with the Pannoramic Midi slide scanner (3D Histech, Budapest, Hungary). The representative images were obtained with the SlideViewer software (3D Histech). The images were analyzed with QuPath (version 0.1.2) software (qupath.github.io) [29]. The proportion of PRRSV-positive cells was determined in units considered to be representative of the sections. We made annotations of 2.37 mm^2^ representative areas in each slide, which is equivalent to 10 high power fields (HPFs) of conventional light microscopy. We used the “positive cell detection” commands from the “analyze”, “cell detection” tab. Then, we retrieved the results with the “show detection measurements” command in the “measure” tab. In addition to the quantity of all detected cells, the number and percentage of positive cells are also immediately given by the software. A detailed step-by-step protocol is provided in Supplementary Material.

### 2.6. PRRSV qRT-PCR

The lung tissue sections (50 mg) were homogenized in a TissueLyser II instrument (Qiagen GmbH, Hilden, Germany) for 3 min. The capped tubes were thoroughly vortexed and centrifuged for phase separation. Then, 200 µL of the supernatants was collected and further processed in a QiaCubeHT instrument using the Cador pathogen Kit for viral nucleic acid purification (Qiagen GmbH), according to the manufacturer´s protocol.

Further, 2 µL of the eluted RNA was used for ORF7 specific RT-PCR using the Luna Onestep RT-PCR Kit (NEB) (forward: TCAACTGTGCCAGTTGCTGG, reverse: TGRGGCTTCTCAGGCTTTTC and 5’Fam -CCCAGCGYCRRCARCCTAGGG Tamra-3’ as probe). The primer sequences were adapted from Egli et al. [30] to fit the sequence of PRRSV-1 strain AUT15-33.

Absolute quantitation of the genome equivalents (GE) was calculated from serially diluted SP6 transcripts of cloned AUT15-33 cDNA of 4872nt. Transcripts were generated using SP6 polymerase from AclI linearized plasmid pLS69. Template DNA was treated with DNAse I (NEB) and RNA was purified using the RNeasy Kit (Qiagen GmbH, Germany). RNA concentration was determined with a Quantus fluorometer and RNA specific fluorescent dye (Promega GmbH, Walldorf, Germany). The number of genome molecules was calculated using the algorithm provided by http://scienceprimer.com/copy-number-calculator-for-realtime-pcr [31]. The calculated GE of this preparation were further confirmed by liquid droplet PCR (Applied Biosystems, Thermo Fisher Scientific Inc., Waltham, MA, USA). qPCR was performed with an Applied Biosystem 7300 instrument (Applied Biosystems, Thermo Fisher Scientific Inc.).

### 2.7. Statistical Analyses

The association between the proportion of the infected cells identified by RNAscope ISH and (1) the log10 of the genome copy numbers detected by qPCR in the same lung lobe, (2) the histological severity score of the same lung lobe, and (3) the overall histological severity of the entire lungs was evaluated by beta regression models with logit link. The calculations were performed in R v4.0.3 [1] using the *betareg* package for the model fitting [2]. We considered an estimate statistically significant when *p* ≤ 0.05.

## 3. Results

### 3.1. Histopathology and In Situ Hybridization

Remarkable differences were observed in the overall lung lesion scores between the challenged and control groups (data not shown as detailed statistical characterization of the lesions was not the aim of the study).

**Figure 1:**
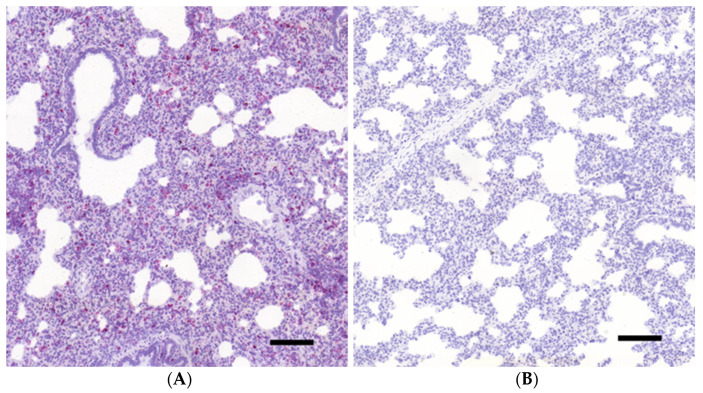
Results of the PPIB labelled positive (**A**) and the bacterial DapB gene labelled negative control (**B**) slides in porcine lung tissue sample examined with RNAscope (12×; bar = 100 µm).

As seen in Figure 1, diffuse, intense, cytoplasmic red staining was seen in the slides hybridized with the *Sus scrofa* PPIB positive control probes, while no apparent signal was present in the DapB probe-treated negative control slides, suggesting proper fixation, tissue integrity, and reaction procedure without a specific signaling.

**Figure 2:**
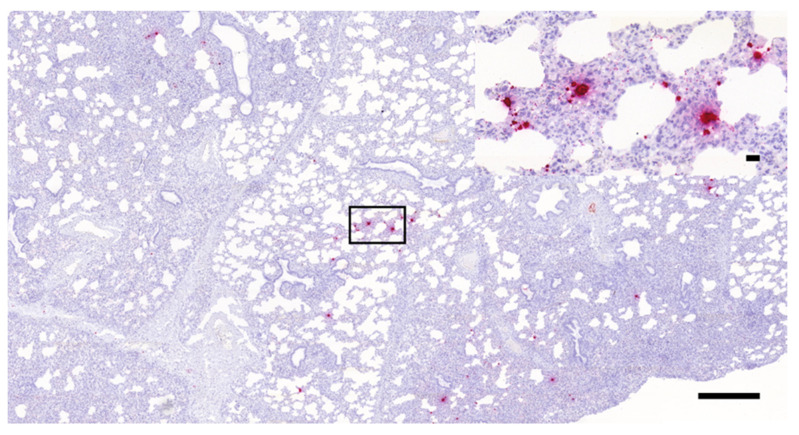
PRRSV-positive porcine lung tissue sample containing small numbers of infected cells (RNAscope ISH, 3×, bar = 500 µm); the inset in the right upper corner shows higher magnification of the indicated area (40×, bar = 20 µm).

**Figure 3:**
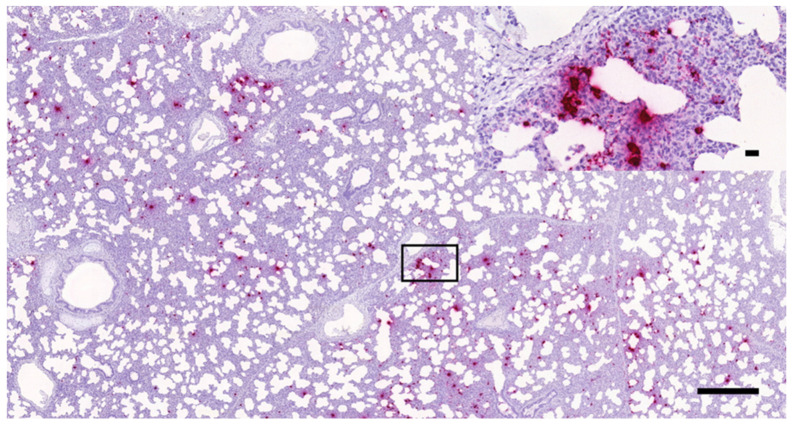
PRRSV-positive porcine lung tissue sample containing moderate numbers of infected cells (RNAscope ISH, 3×, bar = 500 µm); the inset in the right upper corner shows higher magnification of the indicated area (40×, bar = 20 µm).

**Figure 4:**
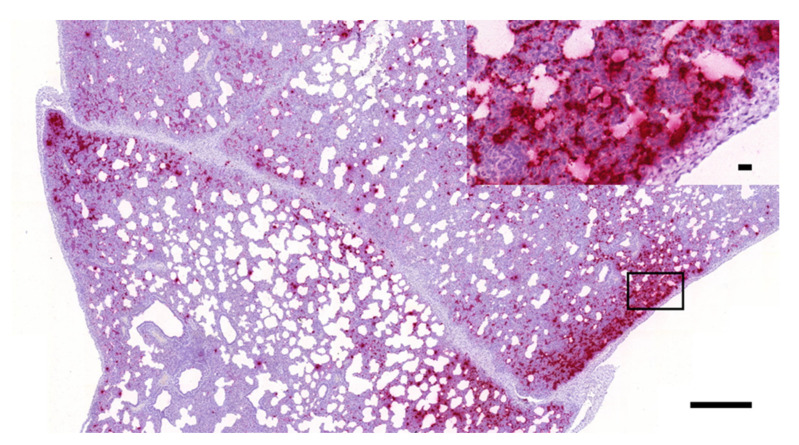
PRRSV-positive porcine lung tissue sample containing high numbers of infected cells (RNAscope ISH, 3×, bar = 500 µm); the inset in the right upper corner shows higher magnification of the indicated area (40×, bar = 20 µm).

As presented in Figure 2, Figure 3 and Figure 4, the presence of PRRSV genome was identified as multifocally distributed, individual, or coalescing red dots on the sections prepared from the lungs of the challenged animals. The number and distribution of the infected cells (mostly with macrophage and alveolar epithelial morphology) were uneven in the sections in most cases. Typically, more positive cells were observed in the areas showing more severe inflammatory lesions, especially where the intralobular septae were wider. Larger numbers of positive cells were mostly observed within these intralobular/interalveolar septae.

### 3.2. qRT-PCR

As expected, no PCR positivity was found in the lung tissue samples of the mock-challenged, negative control animals. All other animals challenged with AUT15-33 were positive for viral genome in their lungs. Table 1 shows the log10 genome copies/g tissue of the samples obtained from the challenged animals ranging from 8.18 to 11.68.

### 3.3. Digital Image Analysis

After the digitalization of the tissue RNAscope slides, QuPath image analysis software was used to determine the number and proportion of infected cells. As expected, no positive cells were identified in the samples obtained from the mock infected animals. In the lung samples of the challenged pigs, the proportion of the infected cells showed marked inter-individual variability, with values ranging from 0.16% to 26.7% on the annotated areas, as reported in Table 1.

### 3.4. Beta Regression

Three separate beta regression models were fit on the proportions of RNAscope ISH labelled PRRSV positive cells calculated digitally on representative annotated areas with (1) the overall histological severity and distribution scores of all seven lobes, (2) the histological severity and distribution scores of the left medial lobe, and (3) the log10 of the genome copy numbers in the left medial lobe detected by qPCR as explanatory variables, respectively (Supplementary Tables S1–S3).

A significant association was found between the proportion of the infected cells and the log10 of the genome copy numbers detected by qPCR in the same lung lobe (pseudo R^2^ = 0.3894, *p* = 0.004, Supplementary Figure S1, Supplementary Table S1). In the case of the overall histological severity, we could not reveal any statistically significant association (pseudo R^2^ = 0.1664, *p* = 0.1806, Supplementary Figure S2, Supplementary Table S2), nor any association with the ISH stained left medial lobe (pseudo R^2^ = 0.1354, *p* = 0.1455, Supplementary Figure S3, Supplementary Table S3).

## 4. Discussion

Direct laboratory diagnosis of PRRSV is primarily based on RT-PCR, and the detection of the viral antigen by immunohistochemistry (IHC) or the viral genome by in situ hybridization in histological slides is usually not part of the routine. These methods—widely used in human and veterinary pathology—allow the examination of the histopathological changes and to observe the amount, the distribution, as well as the cell tropism of the given pathogen within the tissues. These features can be useful to assess the pathological role of PRRSV in natural cases of porcine respiratory disease complex [32], but the methods are more commonly used in experimental settings, where PRRSV is the only pathogen [33].

It is also important to note that there are several pitfalls and difficulties regarding virus detection in histological slides by IHC. In these assays, the accuracy of the results is largely determined by factors such as the concentration and pH of the fixative used, the time elapsed between autopsy and the fixation (autolysis), and the duration of the fixation process, which mostly affect the quality of the samples sent for routine diagnostics, where quality control procedures of the samplings and tissue processing are not implemented like in the case of human pathology [34]. For these reasons, and the high genetic and antigenic variability of the different PRRSV strains, detection of viral antigen by IHC is analytically and diagnostically less sensitive than methods for detection of viral nucleic acids [16].

Different RNA-based in situ hybridization assays have already been developed for the detection of PRRSV including fluorescent (FISH), chromogenic (CISH), and biotinyl tyramide-based (TISH) methods [32,35].

The newly developed RNAscope ISH assay targeting the ORF7 genomic region of the AUT15-33 PRRSV-1 strain successfully detected the virus in the lungs of experimentally challenged animals. In this case, the use of adequate fixative (10% NBF) and proper length of the fixation ensured the integrity of the tissue. The use of RNAscope for the detection of PRRSV has only been reported in the case of PRRSV-2 strains [36,37]. The presence and distribution of the viral genome in the tissue seemed to be overlapping with the severity of the pneumonia; that is, more virus was present in the areas where the interstitial inflammation was more prominent, although no statistically significant association could be found between the proportion of ISH positive cells and severity score of the left medial lung lobe, nor the overall severity of lung lesions. The variable severity and distribution of the lesions between individual pigs could have reduced the power of the comparison, suggesting that a higher number of examined lungs sections may be needed to achieve a statistically significant association.

In order to investigate the applicability of the assay for quantitative measurements, we first used automatic, software-based positive cell counting on slides that were digitalized in a slide scanner instrument. Computational cell counting is widely used in human pathology, especially for Ki67 index and tumor-infiltrating lymphocyte assessments, and it is proved to be comparable to or, in some cases, even more accurate and reproducible than manual counting [26,38,39].

A significant association was found between the proportion of the infected cells and the log10 of the genome copy numbers detected by qPCR in the same lung lobe. An explanation for the varied cell ratios and the simultaneous high copy numbers observed in some cases could be the heterogenous distribution of the lesions/ISH signal density and the two-dimensional nature of the ISH signal quantification compared with qPCR, which analyses the tissue piece as a whole.

The results prove that PRRSV detection by RNAscope ISH and subsequent digital image analysis can be a powerful tool to assess the viral burden in a histological slide, where the tissue structure is also visible. From a pathological point of view, this information is more valuable than a PCR result as the presence of the virus can be directly visualized and evaluated within the lesions.

The price and labor-intensive nature of the RNAscope and the digital image analysis do not allow the method to be used in routine diagnostics yet, but our results proved that it can be a powerful tool for the evaluation of various pathogenicity challenge trials as well as vaccine development studies, where the reduction of the lung inflammation along with decreased positive cell percentage can be an important endpoint to define efficacy.

Our future goal is to validate the assay on different PRRSV positive FFPE samples. As the probes were designed to bind to one of the most conserved regions of the virus’ genome (ORF7) and the method is known to be highly sensitive owing to the numerous smaller probes deployed, there is a great chance that it could be used for the detection of other PRRSV strains as well. The use of the housekeeping positive control probe and the negative control can help to assess the tissue integrity in the cases of FFPE blocks in which the fixation time is unknown, as the latter along with the quality of the fixative (10% NBF) is a critical element of a successful RNAscope reaction.

## 5. Conclusions

By the use of the novel RNAscope ISH assay we have successfully identified and localized PRRSV-1 in the lungs of pigs experimentally infected with the strain AUT15-33. Positive cell counts calculated by digital image analysis on the virtual slides and statistical comparisons with the log10 of the genome copy numbers detected by qPCR in the same lung lobes revealed significant associations between the variables. Our results prove that RNAscope ISH combined with digital image analysis can provide valuable semiquantitative data regarding in situ PRRSV infection of the lungs.

## Figures and Tables

**Table 1:** Percentage of infected cells relative to the 10-based logarithm of genome copies per gram lung tissue.

Animal No.	Proportion Of Infected Cells (%)	log10 Genome Copies/g	L. Med. Severity ^a^	Overall Severity ^b^
19	0.00	0.00	0	0
9	0.00	0.00	0	0
7	0.00	0.00	0	0
4	0.00	0.00	0	0
2	0.00	0.00	0	0
23	0.16	10.09	17	68
22	0.19	10.95	20	143
14	0.20	8.79	10	72
25	0.41	9.80	12	67
21	0.72	9.71	17	101
20	1.04	10.41	16	77
17	1.04	8.18	12	56
5	1.86	10.65	26	180
6	1.88	10.68	19	157
12	2.73	11.46	20	135
3	2.93	10.98	17	101
18	3.86	10.73	23	139
13	3.97	10.66	12	65
1	4.24	11.25	17	107
15	4.29	10.91	21	115
11	5.60	11.00	11	110
24	5.79	10.70	25	144
8	8.25	10.93	19	110
10	9.02	10.99	20	136
16	26.70	11.68	21	137

^a^ Severity and distribution scores of the left medial lung lobe; ^b^ overall severity and distribution scores of all the lobes.

## Data Availability

The data presented in this study are available in this article and its Supplementary Materials.

## Supplementary Materials

The following are available online at https://www.mdpi.com/article/10.3390/vetsci8100235/s1: Quantification of PRRSV-infected cells labeled by *in situ* hybridization in porcine lung tissues with QuPath software (version 0.1.2). Table S1: Parameter estimates of the beta regression model fitted on the proportion of RNAscope ISH labelled PRRSV positive cells and the log10 of the genome copy numbers detected by qPCR; Table S2: Parameter estimates of the beta regression model fitted on the proportion of RNAscope ISH labelled PRRSV positive cells and overall histological severity; Table S3: Parameter estimates of the beta regression model fitted on the proportion of RNAscope ISH labelled PRRSV positive cells and the scores obtained for the ISH stained left medial lobe; Figure S1: Proportion of the PRRSV ISH positive cells and the log10 of the genome copy numbers detected by qPCR; Figure S2: Proportion of the PRRSV ISH positive cells and the overall histological severity; Figure S3: Proportion of the PRRSV ISH positive cells and the scores obtained for the ISH stained left medial lobe.
