# Supplementary material for: In Situ Hybridization of PRRSV-1 Combined with Digital Image Analysis in Lung Tissues of Pigs Challenged with PRRSV-1
Source: Vet Sci. 2021 Oct 15;8(10):235. doi: 10.3390/vetsci8100235 (PMC8540710; doi:10.3390/vetsci8100235)

Quantification of PRRSV-infected cells labeled by *in situ* hybridization in porcine lung tissues with QuPath software (version 0.1.2)

1. Open QuPath.
2. Create a new project, optimally in an empty folder with the name of the project or experiment.
3. Add and import images with the default settings.
4. Double click on the image.
5. Set image type to Brightfield.
6. Create an annotation of 2,37mm^2 representative of the section. If the annotations window is open, the size of the area can be seen in parallel with the selection.
7. Run the positive cell detection from the cell detection command within the analyze tab. Use the default settings.
8. Retrieve the results with the show detection measurements or show annotation measurements command from the measure tab.
9. Repeat for other added slides as appropriate.

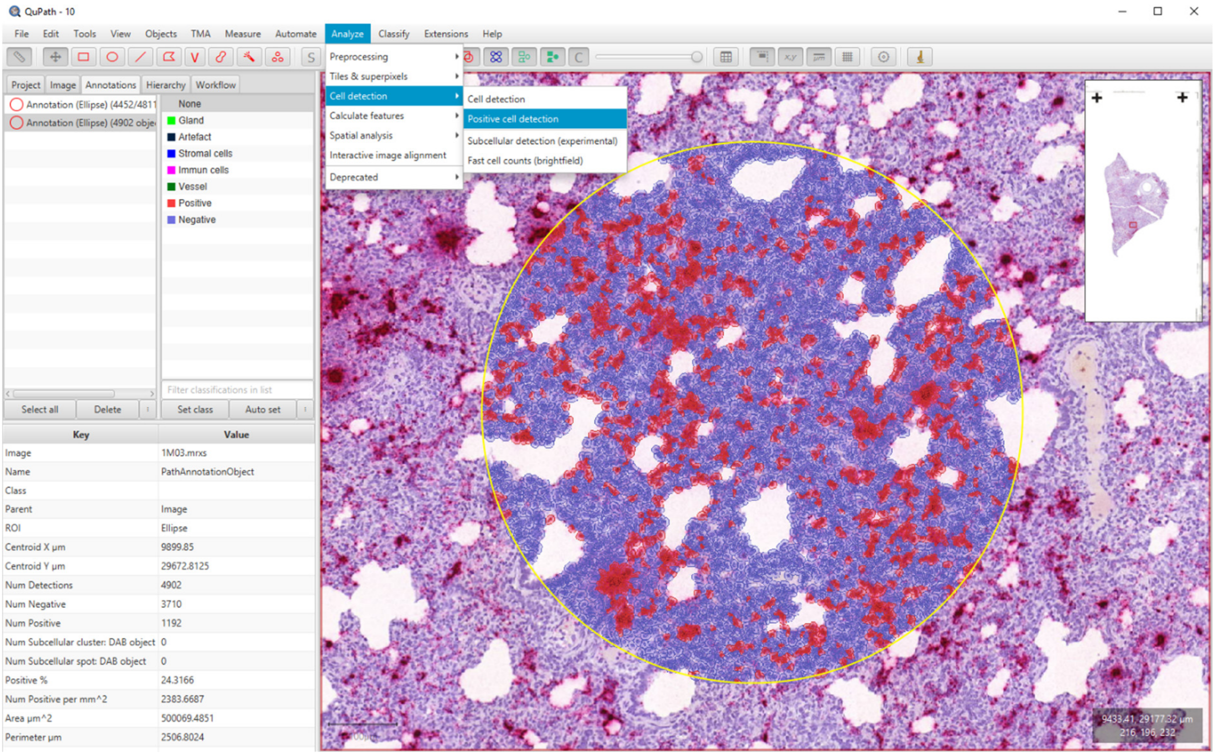

Supplementary Table S1
Parameter estimates of the beta regression model fitted on the proportion of RNAscope ISH labelled PRRSV positive cells and the log10 of the genome copy numbers detected by qPCR. The estimates and their standard error (*Estimate (SE)*) are backtransformed from the logarithm scale. They represent the fold change in the ratio of the positive cells compared to the negative ones, when
the log10 of the genome copy numbers detected by qPCR is raised by 1 unit.

Coefficients	Estimate (SE)	p value
Intercept	4.5155e-06 (13.8507)	<0.0001
log10 Genome Copy	2.3586 (1.2725)	0.0004

Supplementary Table S2
Parameter estimates of the beta regression model fitted on the proportion of RNAscope ISH labelled PRRSV positive cells and overall histological severity. The estimates and their standard error (*Estimate (SE)*) are backtransformed from the logarithm scale. They represent the fold change in the ratio of the positive cells compared to the negative ones, when the overall histological severity is raised by 1 unit.

Coefficients	Estimate (SE)	p value
Intercept	0.0193 (2.0069)	<0.0001
Overall severity	1.0074 (1.0055)	0.1806

Supplementary Table S3
Parameter estimates of the beta regression model fitted on the proportion of RNAscope ISH labelled PRRSV positive cells and the scores obtained for the ISH stained left medial lobe. The estimates and their standard error (*Estimate (SE)*) are backtransformed from the logarithm scale. They represent the fold change in the ratio of the positive cells compared to the negative ones, when the scores obtained for the ISH stained left medial lobe is raised by 1 unit.

Coefficients	Estimate (SE)	p value
Intercept	0.0145 (2.3077)	<0.0001
Medial lobe severity	1.064 (1.0435)	0.1455

Supplementay Figure S1

Proportion of the PRRSV ISH positive cells and the log10 of the genome copy numbers detected by qPCR. The red line indicates the fitted curve from the respective beta regression model.

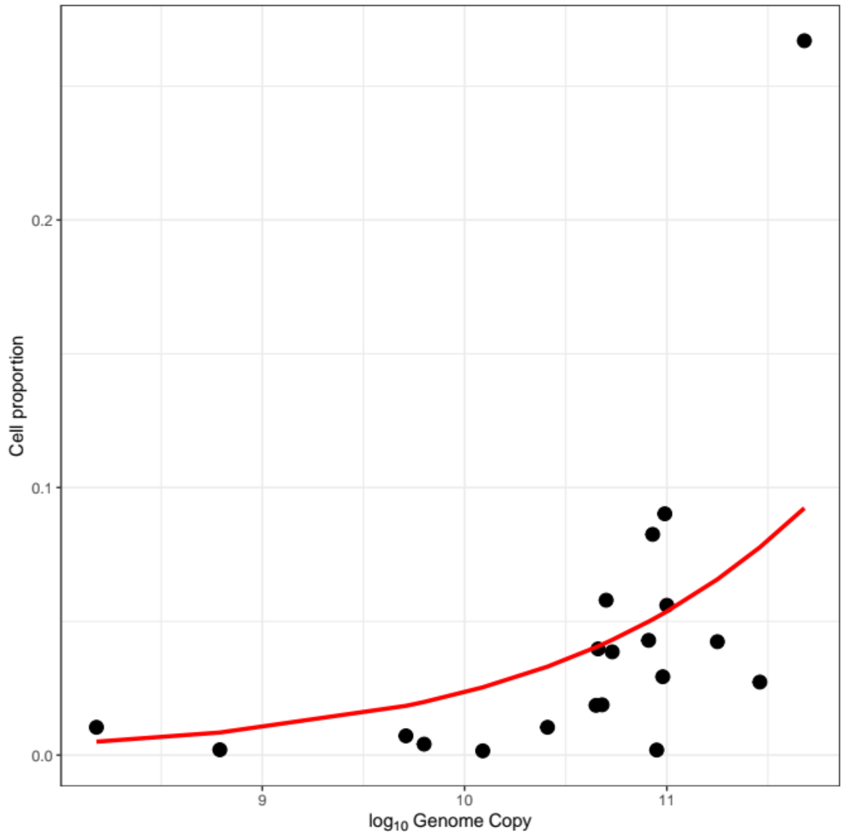

Supplementay Figure S2

Proportion of the PRRSV ISH positive cells and the overall histological severity. The red line indicates the fitted curve from the respective beta regression model.

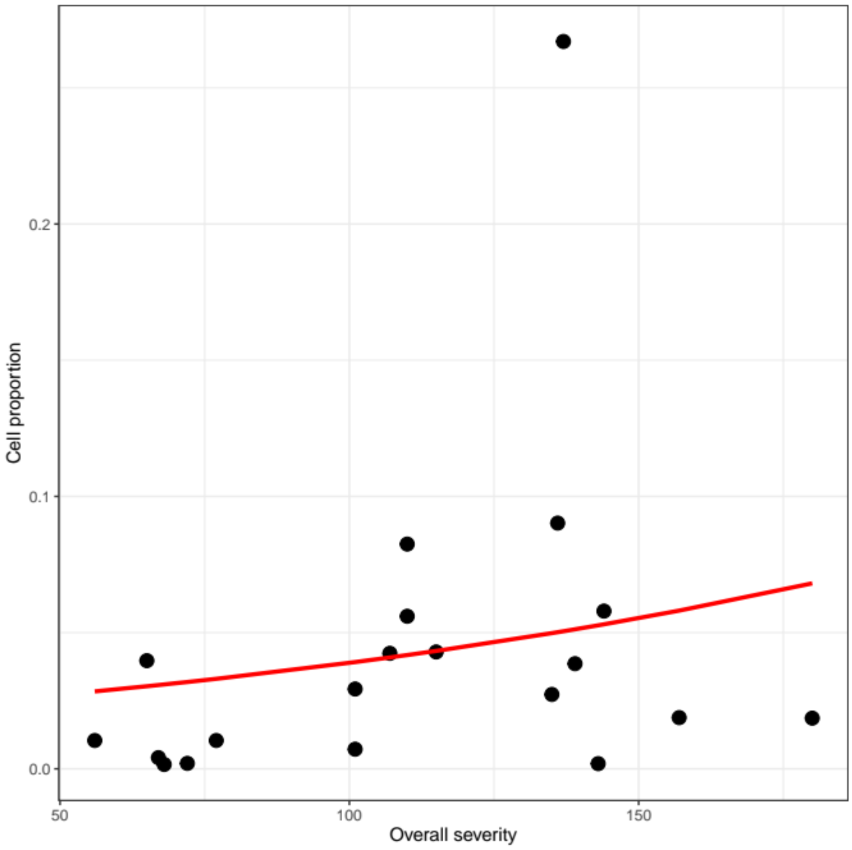

Supplementay Figure S3

Proportion of the PRRSV ISH positive cells and the scores obtained for the ISH stained left medial lobe. The red line indicates the fitted curve from the respective be

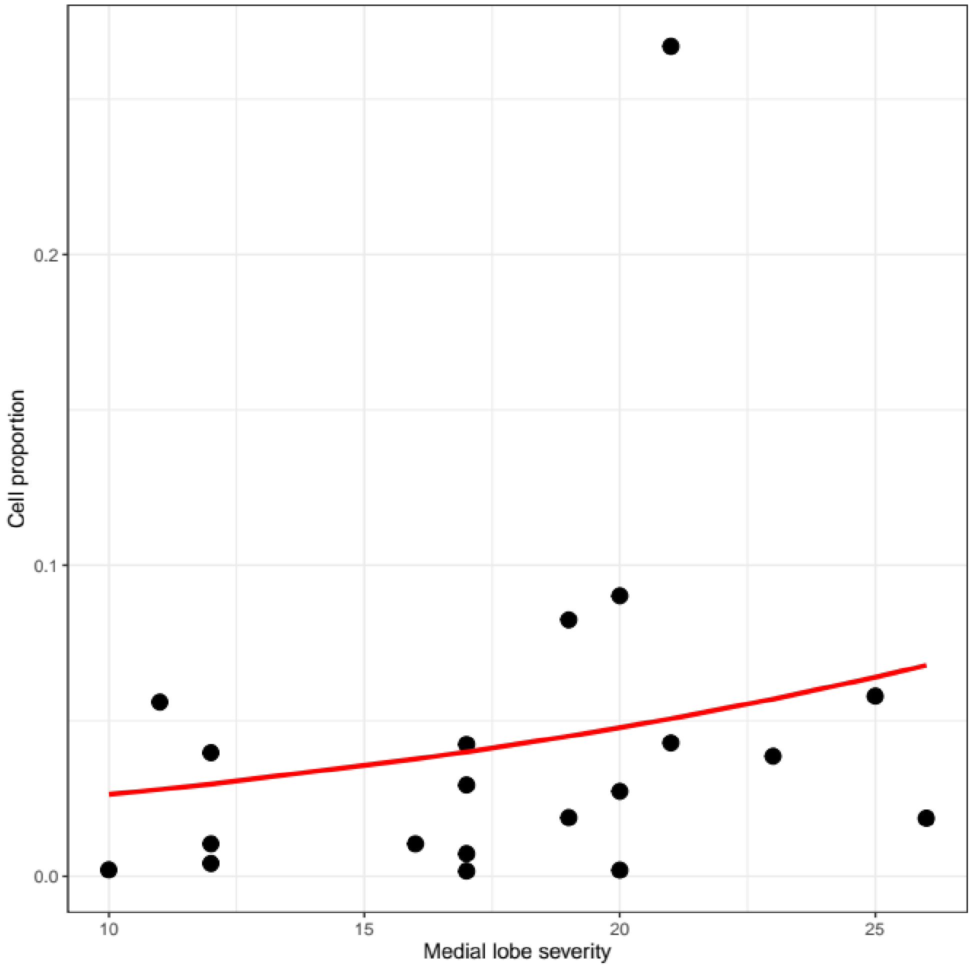

Supplement: Supplementary file 1 [file vetsci-08-00235-s001.zip › vetsci-1387892-supplementary.pdf]
